# Study of Macrophage Activity in Cats with FIP and Naturally FCoV-Shedding Healthy Cats

**DOI:** 10.3390/pathogens13060437

**Published:** 2024-05-22

**Authors:** Sara Mangiaterra, Alessandra Gavazza, Lucia Biagini, Giacomo Rossi

**Affiliations:** School of Biosciences and Veterinary Medicine, University of Camerino, 62032 Camerino, Italy; alessandra.gavazza@unicam.it (A.G.); lucia.biagini@unicam.it (L.B.); giacomo.rossi@unicam.it (G.R.)

**Keywords:** cats, macrophage, phagocytosis, respiratory burst, FIP, FCoV

## Abstract

Coronavirus frequently infects humans and animals, showing the ability to recombine and cross over to different species. Cats can be considered a model for studying coronavirus infection, in which feline coronavirus (FCoV) represents a major enteric pathogen related to gastroenteric disease. In this animal, the virus can acquire tropism for macrophage cells, leading to a deadly disease called feline infectious peritonitis (FIP). In this study, monocyte-derived macrophages were isolated by CD14-positive selection in venous whole blood from 26 cats with FIP and 32 FCoV-positive healthy cats. Phagocytosis and respiratory burst activities were investigated and compared between the groups. This is the first study comparing macrophage activity in cats affected by FIP and healthy cats positive for FCoV infection. Our results showed that in cats with FIP, the phagocytic and respiratory burst activities were significantly lower. Our results support the possible role of host immunity in Coronaviridae pathogenesis in cats, supporting future research on the immune defense against this systemic disease.

## 1. Introduction

Feline coronavirus (FCoV) is a major enteric pathogen of the Felidae family with a worldwide distribution [1,2]. In cats, FCoV replicates in the intestines and can spread by oral–fecal transmission but not yet understood changes can give rise to mutants associated with a deadly disease called feline infectious peritonitis (FIP) [2]. In FIP, there are two clinical forms, which can occur separately or coexist [3]. During FIP infection, macrophage cells release proinflammatory cytokines including IL-1α, IL-1β, IL-6, and tumor necrosis factor, and along with severe T cell depletion, this causes a “cytokine storm”, akin to that seen in COVID-19 infection [4,5,6]. The effusive FIP is characterized by pyogranuloma and the infection is considered a distinct form of vasculitis [2]. This form is particularly prevalent in the abdomen, covering the serosal surfaces of organs; fibrin and protein-rich fluid are also deposited within and around the lesions and necrosis is often evident [5]. The “dry or non-effusive” form is characterized by a wide variety of clinical signs involving the eyes, brain, or other organs of the body, leading to a variety of clinical signs, but up to 30% of affected cats present with neurological involvement [7,8,9]. Both neurological and generalized FIP may present first as a nonspecific illness, with clinical signs including fever, weight loss, and lethargy. Mesenteric lymphadenopathy and irregular splenic and renal surfaces are commonly detected on abdominal examination of cats with neurological FIP. The pathogenesis of FIP is still not fully understood; some studies suggested that the responses of macrophages to the virus and the depletion of CD4+ and CD8+ T lymphocytes are key points for the virus–host interactions [5,6,7,8,9,10,11]. Studies have suggested that the mutation of FECV leads to pathogenic FIPV, and consequently, tropism for monocytes/macrophages causes the disease, leading to typical immunopathological damage [12]. Macrophage cells release proinflammatory cytokines including IL-1α, IL-1β, IL-6, and tumor necrosis factor (TNF), akin to apoptosis and cytokine storms [13]. Even though SARS-CoV-2 does not target monocytes, the role of antibodies and the immune complexes is not as well-defined as in feline infectious peritonitis. Nevertheless, as is seen during septic shock, and even during SARS-CoV2, the effect of the activation of the cholinergic anti-inflammatory pathway (CAP) by α7 subunit-containing nicotinic receptor (α7nAChR) on macrophages can lead to a much less severe response of the host immune system, with a significant reduction in mortality [14]. In COVID-19, inflammation is induced by a cytokine storm characterized by T cell cytokines such as IFNγ and IL-3 that activate macrophages to produce IL-1, IL-6, and TNFα [15]. During SAR-CoV-2 infection, pulmonary macrophages derived from infiltrating monocytes are hyperactivated, resulting in the recruitment of cytotoxic cells, exacerbating damage and leading to the cytokine storm [16]. In FIP and COVID-19 disease, monocyte-derived macrophages have a key role in the immunopathology and their dysregulated functions can lead to organ damage and induce multi-organ failure syndrome [17,18]. In the pathogenesis of both infections, cytokine storms have been implicated with the over-expression of inflammatory cytokines; during feline infectious peritonitis, this is due to the activation of monocytes and macrophages, while in COVID-19, the link to macrophages and monocytes is not fully understood [17,18,19]. Different methods have been employed to isolate monocytes from peripheral blood and to study their activity. Phagocytosis can be assessed by microscopy to discern between engulfed particles and those that are not engulfed by flow cytometry or the fluorometric plate-based approach [20]. Respiratory burst is characterized by the rapid release of reactive oxygen species. It is used in immunological studies, and it is often assessed by plate-based colorimetric assays or flow cytometry [20]. In this study, peripheral blood monocytes from 26 cats with FIP were cultured, and phagocytosis and respiratory burst activity was assessed and compared with that of macrophages derived from 32 healthy cats with an intestinal, unapparent form of FCoV infection.

## 2. Materials and Methods

To assess the biological variation and repeatability of the described methods, each assay was conducted in separate trials, each consisting of one sample obtained from different subjects for each trial. Ethical review and approval were not required for this study because this case report referred to a spontaneous medical condition of client-owned cats; samples used were those collected for medical purposes. Written informed consent was obtained from the owners before enrolling their animals in the study. This study consisted of a study group of cats affected by FIP and a control group of naturally FCoV-shedding healthy cats. Study group population consisted of 26 cats diagnosed with feline infectious peritonitis, of which 14 had the clinical “wet form” and 12 had the “dry form”. The diagnosis for the wet form was made by RT-PCR examination on a sample of abdominal effusion, which was positive for FIP. The diagnosis for the clinical dry form was made by PCR assay on abdominal lymph node biopsy or cerebrospinal fluid (CSF). Control group population consisted of 32 healthy cats positive for FCoV in fecal samples by RT-PCR. All subjects tested negative for other infectious diseases (feline immunodeficiency virus or FIV, feline leukemia virus or FeLV, and *Toxoplasma* spp).

### 2.1. Monocyte Phagocytosis Activity

In subjects of both groups, monocytes were isolated from blood by CD14-positive selection with QuadroMAcs separator, in accordance with the manufacturer’s instructions (Miltenyi Biotec). PBMCs for CD14-positive selection were diluted to 1 × 10^8^ cells/mL Leibovitz’s L-15 cell medium supplemented with 10% FCS, 100 U/100 µg/mL penicillin/streptomycin (ThermoFisher Scientific). A volume of 200 μL of monocyte suspension with and without phorbol 12-myristate 13-acetate (PMA) was distributed in duplicate on polarized slides. Slides were placed in a 37 °C humidified incubator to recover overnight. Slides were placed in a humidified incubator at 37 °C for 90 min to allow phagocytosis. After incubating for 24 h at 37 °C, the medium was carefully aspirated and then 200 μL liposomes was resuspended and added to the cell culture. These cells were also incubated at 37 °C for 4 h to measure internalization of the liposomes. After 3 × PBS washes, slides were then analyzed with C2 Plus confocal laser scanning microscope (Nikon Instruments, Firenze, Italy). Optimized emission detection bandwidths were configured by Zeiss Zen control software. Images were processed using NIS Element Imaging Software (Nikon Instruments, Firenze, Italy). Phagocytosis was measured by counting, microscopically, the number of ingested liposomes within the macrophages. For this purpose, three randomly selected fields were evaluated at 40 × HPFs per sample, and a total cell count was performed, recording those that were fluorescent from having engulfed liposomes, and counting the average number of phagocytized liposomes per cell.

### 2.2. Monocyte Respiratory Burst Activity

In subjects of both groups, monocytes were isolated by CD14-positive selection with QuadroMAcs separator, in accordance with the manufacturer’s instructions (Miltenyi Biotec). To assess the respiratory burst activity, PBMCs for CD14-positive selection were diluted to 1 × 10^8^ cells/mL Leibovitz’s L-15 cell medium supplemented with 10% FCS, 100 U/100 µg/mL penicillin/streptomycin (ThermoFisher Scientific). Cell viability was evaluated by MTT assay and was greater than 98%. Isolated cells were cultured in 96-well plates at 37 °C with 5% CO2 for 24 h, allowing the cells to recover overnight. A volume of 50 µL of medium was removed from the top of each well. Cells should have settled to the bottom and therefore not be removed from the plate, as reported by Hampton et al. [21]. After vigorously vortexing, 5 µL of nitroblue tetrazolium (NBT) and 0.5 µL of phorbol 12-myristate 13-acetate (PMA) were added in triplicate to each sample and then the plate was returned immediately to the incubator for 1 h. Following incubation, 100 µL 70% methanol (MeOH) was added to each well and washed two more times. The plate was allowed to air dry at room temperature. After the plate was completely dry, 120 µL of 2M KOH was added to each well followed by 140 µL of dimethyl sulfoxide (DMSO) and mixed. The absorbance of the solution on a standard plate reader at 620 nm is measured.

### 2.3. Statistical Analysis

A two-way analysis of variance (ANOVA) with Sidak’s multiple comparisons test was used to analyze differences between phagocytic and non-phagocytic cell percentage between FIP+ and healthy FCoV-shedding cats (FIP-) to investigate differences in PMA-stimulated respiratory burst activity between the same two categories of cats. Moreover, a Mann–Whitney test was used to compare differences within FIP+ and FIP- groups both for phagocytic percentage and respiratory burst activity. All statistical analyses were performed with GraphPad Prism 8 (GraphPad Software Inc., San Diego, CA, USA). *p* < 0.05 was considered significant.

## 3. Results

**Figure 1:**
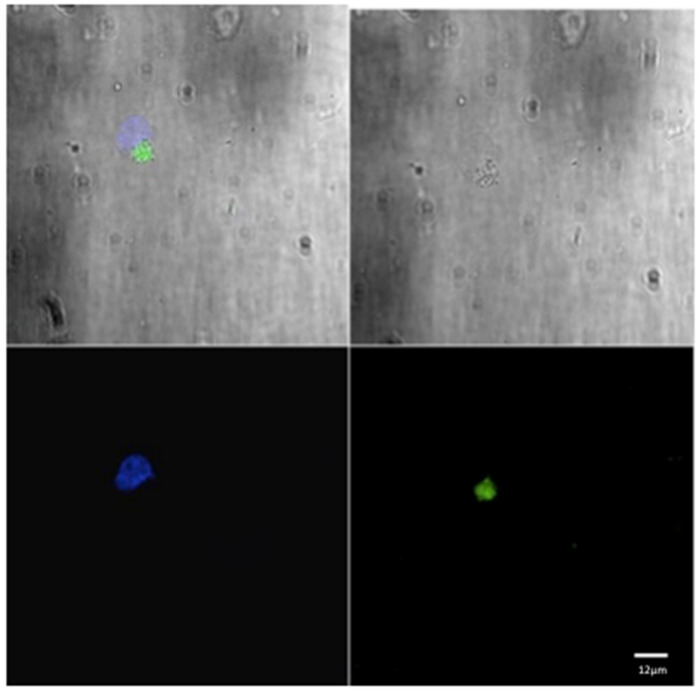
Confocal laser scanning microscope images of liposome particles engulfed by monocyte-derived macrophages. Note the nuclear (DAPI-stained) material of the macrophages (blue) and the intra-cytoplasmatic liposomes (green) detected by confocal microscope. Scale bars 12 µm.

**Figure 2:**
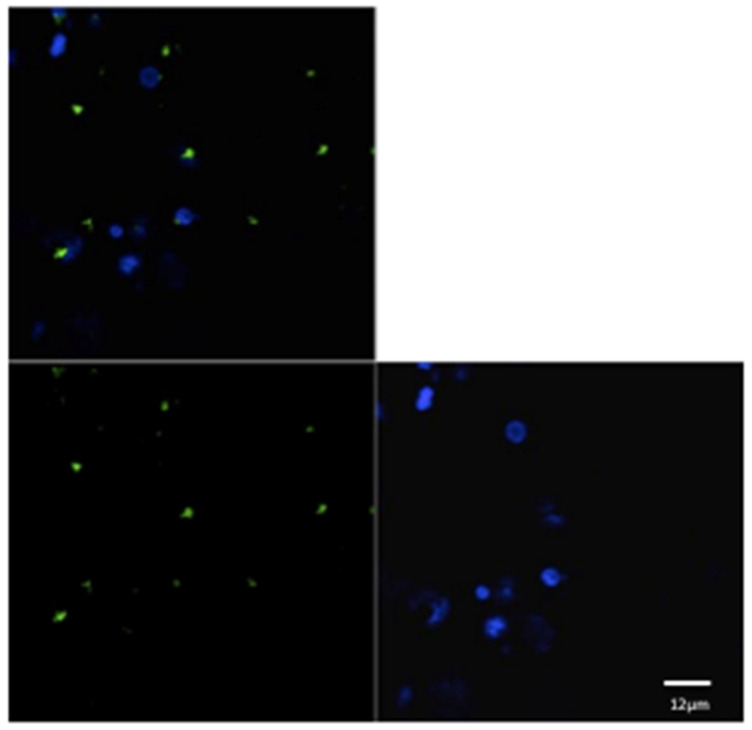
Confocal laser scanning microscope images of liposome particles engulfed by monocyte-derived macrophages. Note the nuclear (DAPI-stained) material of the macrophages (blue) and the intra-cytoplasmatic liposomes (green) detected by confocal microscope. Scale bars 12 µm.

For each animal in both groups, three microscopic fields were examined to evaluate the phagocytic activity (Figure 1 and Figure 2). The mean of the number of phagocytic cells (macrophages) undergoing phagocytosis was evaluated.

The mean value of phagocytic cell activity (Table 1) and respiratory burst (Table 2) of the macrophages belonging to the group of cats with the FIP form of FCoV was significantly lower when compared with that of the healthy cats with an intestinal, unapparent form of FCoV infection (*p* < 0.0001).

**Figure 3:**
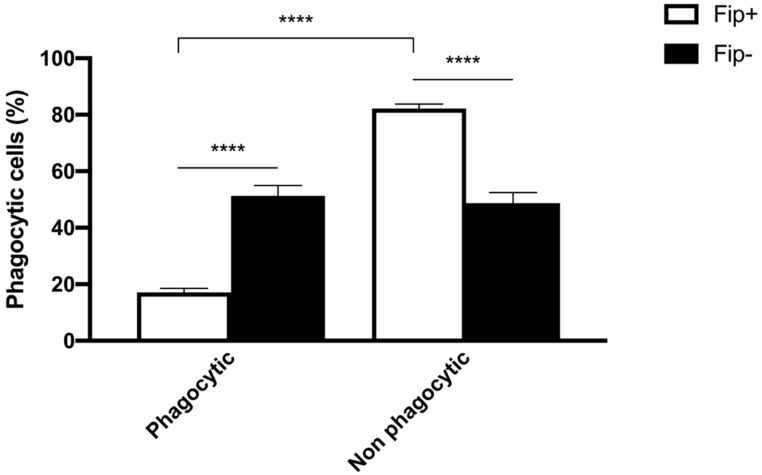
Schematic representation of phagocytic and non-phagocytic cell percentage in FIP+ and FIP– groups. **** *p* < 0.0001.

**Figure 4:**
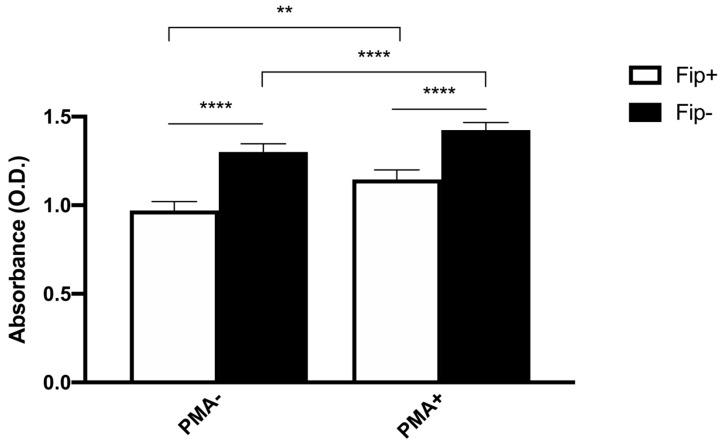
Schematic representation of absorbance in non-stimulated (PMA-) and stimulated (PMA+) macrophages in FIP+ and FIP– groups. ** *p* < 0.01, **** *p* < 0.0001.

An ANOVA with Sidak’s multiple comparisons test was used to analyze the differences between the phagocytic and non-phagocytic cell percentage between the FIP+ and FIP- cats (Figure 3). The results reached significance. The absorbance of the cultured cells from the FIP+ and FIP- cats without PMA and stimulated with PMA, respectively, was measured on a standard plate reader at 620 nm. The (mean ± standard deviation) of the absorbance calculated without PMA stimulation in the FIP+ group was lower in comparison to the absorbance calculated in the FIP- group (1.3 ± 0.26) (*p* < 0.01). The (mean ± standard deviation) of the absorbance calculated after the PMA stimulation of the cells belonging to the FIP + group was lower (1.14± 0.27) than the absorbance calculated for the cells from the cats of the control group (1.4 ± 0.23) (*p* < 0.0001). An ANOVA with Sidak’s multiple comparisons test was used to analyze the differences in the PMA-stimulated respiratory burst activity between the FIP+ and FIP- cats. The results reached significance (Figure 4).

## 4. Discussion

An inadequate or altered response of a host’s immune system could characterize a disease [22]. In this perspective, even the fundamental concept that “the organism causes the disease” should probably be abandoned in light of the multi-stage pathogenesis of all diseases. Modern medicine is changing the vocabulary of infections; familiar terms such as primary pathogen, opportunistic infection, and immunocompetent patient need to be re-examined in light of what we have learned about hosts and their unique way of responding to pathogens. Macrophages are considered professional phagocytes for pathogen clearance, representing a formidable weapon against all pathogens. In this study, a method to test macrophages’ functional capacity ex vivo is described. This method offers several advantages due to its speed and simplicity; with this assay, cellular phagocytic capacity can be quantified. The experiments reported demonstrate that in FIP+ cats, monocyte-derived macrophages exhibit significantly lower phagocytic activity than FCoV healthy cats. In physiologic conditions, after the inflammatory response, M1-like macrophages switch to the M2-like phenotype to initiate the resolution of inflammation [23]. M1 macrophages constitute the first line of defense against intracellular pathogens and promote Th1 polarization of CD4+ lymphocytes by the interleukin IL-12 [24]. M1 macrophages differentiate under the influence of IFN-γ and/or LPS and are characterized by high levels of pro-inflammatory cytokines, reactive oxygen intermediates and nitric oxide synthase-2 [25]. In contrast, M2 macrophages have been initially identified under the influence of IL-4 and IL-13 produced during the Th2-polarized response [26]. The interaction between pathogens and the host immune response is characterized by various strategies. M2 macrophages appear to be a “favorable condition” for the long-term persistence of intracellular pathogens. In humans, studies suggest that the pathogenesis of SARS-CoV2 infection, especially in cases with acute respiratory distress syndrome (ARDS), depends on phenotypes and functionalities of monocytes and monocyte-derived macrophages [27]. The present results demonstrate that these assays can be used to study immune function and to detect perturbation of cellular function in animals with immunological impairment. In conclusion, monocytes are considered an intermediate stage between bone marrow precursors and tissue macrophages. Circulating monocytes exhibit important effector activities in homeostasis and repair functions during infections. Our results suggest that in FIP+ cats, the macrophage is deficient in the recognition and elimination of the virus, with a reduction in phagocytic and respiratory burst activity. This supports the hypothesis that, in FCoV infection, a small population of susceptible cats lack the ability to destroy the virus, developing the deadly form of the disease, or FIP. According to our results, the individual host responses play an important role in disease pathogenesis. To date, few studies on macrophage characterization in FIP cats have been reported, but no comparisons have been made with naturally FCoV-shedding healthy cats. Further studies will be required to investigate the actual activity of macrophages towards FCoV and possible therapeutic approaches towards establishing a subjective immune system that does not target this pathogen.

## Figures and Tables

**Table 1:** Mean number of phagocytic and non-phagocytic cells evaluated in FIP+ cats.

Cat (FIP+)	Phagocytic Cells (%)	Non-Phagocytic Cells (%)
1	13	87
2	6	94
3	10	90
4	15	85
5	20	80
6	18	82
7	23	77
8	13	87
9	5	95
10	10	90
11	8	92
12	20	80
13	15	85
14	15	85
15	25	65
16	15	78
17	12	88
18	21	79
19	19	81
20	15	85
21	25	75
22	25	75
23	12	88
24	22	78
25	30	70
26	33	67
Mean	17.1	82.9

**Table 2:** Absorbance measured in FIP+ cats without PMA and stimulated with PMA.

Cat (FIP+)	PMA-	PMA+
**1**	0.517	0.761
**2**	0.671	0.744
**3**	1.213	1.397
**4**	0.493	0.573
**5**	0.578	0.69
**6**	0.507	0.646
**7**	1.26	1.37
**8**	1.47	1.56
**9**	1.11	1.44
**10**	1.16	1.32
**11**	0.96	1.2
**12**	0.87	1.15
**13**	1.15	1.41
**14**	1.16	1.47
**15**	0.835	0.859
**16**	1.156	1.341
**17**	1.086	1.219
**18**	1.145	1.219
**19**	1.09	1.124
**20**	0.771	0.992
**21**	0.953	1.112
**22**	1.160	1.32
**23**	0.798	1.04
**24**	1.060	1.192
**25**	1.030	1.23
**26**	1.035	1.39
**Mean**	0.1	1.14

## Data Availability

All data are available in the main text.

## References

[1] Sykes J.E. (2014). Feline Coronavirus Infection. Canine and Feline Infectious Diseases.

[2] Pedersen N.C., Allen C.E., Lyons L.A. (2008). Pathogenesis of feline enteric coronavirus infection. J. Feline Med. Surg..

[3] Tsai H.-Y., Chueh L.-L., Lin C.-N., Su B.-L. (2011). Clinicopathological findings and disease staging of feline infectious peritonitis: 51 cases from 2003 to 2009 in Taiwan. J. Feline Med. Surg..

[4] Dhama K., Patel S.K., Pathak M., Yatoo M.I., Tiwari R., Malik Y.S., Singh R., Sah R., Rabaan A.A., Bonilla-Aldana D.K. (2020). An update on SARS-CoV-2/COVID-19 with particular reference to its clinical pathology, pathogenesis, immunopathology and mitigation strategies. Travel Med. Infect. Dis..

[5] Kipar A., Meli M.L., Failing K., Euler T., Gomes-Keller M.A., Schwartz D., Lutz H., Reinacher M. (2006). Natural feline coronavirus infection: Differences in cytokine patterns in association with the outcome of infection. Veter. Immunol. Immunopathol..

[6] Paltrinieri S., Giordano A., Stranieri A., Lauzi S. (2020). Feline infectious peritonitis (FIP) and coronavirus disease 19 (COVID-19): Are they similar?. Transbound. Emerg. Dis..

[7] Felten S., Hartmann K. (2019). Diagnosis of Feline Infectoius Peritonitis: A review of the current literature. Viruses.

[8] Foley J.E., Leutenegger C. (2001). A Review of Coronavirus Infection in the Central Nervous System of Cats and Mice. J. Veter. Intern. Med..

[9] Kent M. (2009). The cat with neurological manifestations of systemic disease. Key conditions impacting on the CNS. J. Feline Med. Surg..

[10] Dewerchin H.L., Cornelissen E., Nauwynck H.J. (2005). Replication of feline coronaviruses in peripheral blood monocytes. Arch. Virol..

[11] Pedersen N.C. (2009). A review of feline infectious peritonitis virus infection: 1963–2008. J. Feline Med. Surg..

[12] Vogel L., Van der Lubben M., Lintelo E.G.T., Bekker C.P., Geerts T., Schuijff L.S., Grinwis G.C., Egberink H.F., Rottier P.J. (2010). Pathogenic characteristics of persistent feline enteric coronavirus infection in cats. Veter. Res..

[13] Malbon A.J., Fonfara S., Meli M.L., Hahn S., Egberink H., Kipar A. (2019). Feline Infectious Peritonitis as a Systemic Inflammatory Disease: Contribution of Liver and Heart to the Pathogenesis. Viruses.

[14] Qin Z., Xiang K., Su D.-F., Sun Y., Liu X. (2021). Activation of the Cholinergic Anti-Inflammatory Pathway as a Novel Therapeutic Strategy for COVID-19. Front. Immunol..

[15] Wang W., Ye L., Ye L., Li B., Gao B., Zeng Y., Kong L., Fang X., Zheng H., Wu Z. (2007). Up-regulation of IL-6 and TNF-α induced by SARS-coronavirus spike protein in murine macrophages via NF-κB pathway. Virus Res..

[16] Knoll R., Schultze J.L., Schulte-Schrepping J. (2021). Monocytes and Macrophages in COVID-19. Front. Immunol..

[17] Gómez-Rial J., Rivero-Calle I., Salas A., Martinón-Torres F. (2020). Role of Monocytes/Macrophages in Covid-19 Pathogenesis: Implications for Therapy. Infect. Drug Resist..

[18] Meidaninikjeh S., Sabouni N., Marzouni H.Z., Bengar S., Khalili A., Jafari R. (2021). Monocytes and macrophages in COVID-19: Friends and foes. Life Sci..

[19] Merad M., Martin J.C. (2020). Pathological inflammation in patients with COVID-19: A key role for monocytes and macrophages. Nat. Rev. Immunol..

[20] Platt N., Fineran P. (2015). Measuring the phagocytic activity of cells. Methods Cell Biol..

[21] Hampton L.M.T., Jeffries M.K.S., Venables B.J. (2020). A practical guide for assessing respiratory burst and phagocytic cell activity in the fathead minnow, an emerging model for immunotoxicity. MethodsX.

[22] Inglis T.J.J. (2007). Principia ætiologica: Taking causality beyond Koch’s postulates. J. Med. Microbiol..

[23] Atri C., Guerfali F.Z., Laouini D. (2018). Role of Human Macrophage Polarization in Inflammation during Infectious Diseases. Int. J. Mol. Sci..

[24] Fadok V.A., Bratton D.L., Konowal A., Freed P.W., Westcott J.Y., Henson P.M. (1998). Macrophages that have ingested apoptotic cells in vitro inhibit proinflammatory cytokine production through autocrine/paracrine mechanisms involving TGF-beta, PGE2, and PAF. J. Clin. Investig..

[25] Muraille E., Leo O., Moser M. (2014). Th1/Th2 Paradigm Extended: Macrophage Polarization as an Unappreciated Pathogen-Driven Escape Mechanism?. Front. Immunol..

[26] Martinez F.O., Combes T.W., Orsenigo F., Gordon S. (2020). Monocyte activation in systemic Covid-19 infection: Assay and rationale. EBioMedicine.

[27] Kosyreva A., Dzhalilova D., Lokhonina A., Vishnyakova P., Fatkhudinov T. (2021). The Role of Macrophages in the Pathogenesis of SARS-CoV-2-Associated Acute Respiratory Distress Syndrome. Front. Immunol..

